# Generative AI for brain image computing and brain network computing: a review

**DOI:** 10.3389/fnins.2023.1203104

**Published:** 2023-06-13

**Authors:** Changwei Gong, Changhong Jing, Xuhang Chen, Chi Man Pun, Guoli Huang, Ashirbani Saha, Martin Nieuwoudt, Han-Xiong Li, Yong Hu, Shuqiang Wang

**Affiliations:** ^1^Shenzhen Institutes of Advanced Technology, Chinese Academy of Sciences, Shenzhen, China; ^2^Department of Computer Science, University of Chinese Academy of Sciences, Beijing, China; ^3^Department of Computer and Information Science, University of Macau, Macau, China; ^4^Department of Oncology and School of Biomedical Engineering, McMaster University, Hamilton, ON, Canada; ^5^Institute for Biomedical Engineering, Stellenbosch University, Stellenbosch, South Africa; ^6^Department of Systems Engineering, City University of Hong Kong, Hong Kong, China; ^7^Department of Orthopaedics and Traumatology, The University of Hong Kong, Hong Kong, China

**Keywords:** generative models, brain imaging, brain network, diffusion model, generative adversarial network, variational autoencoder

## Abstract

Recent years have witnessed a significant advancement in brain imaging techniques that offer a non-invasive approach to mapping the structure and function of the brain. Concurrently, generative artificial intelligence (AI) has experienced substantial growth, involving using existing data to create new content with a similar underlying pattern to real-world data. The integration of these two domains, generative AI in neuroimaging, presents a promising avenue for exploring various fields of brain imaging and brain network computing, particularly in the areas of extracting spatiotemporal brain features and reconstructing the topological connectivity of brain networks. Therefore, this study reviewed the advanced models, tasks, challenges, and prospects of brain imaging and brain network computing techniques and intends to provide a comprehensive picture of current generative AI techniques in brain imaging. This review is focused on novel methodological approaches and applications of related new methods. It discussed fundamental theories and algorithms of four classic generative models and provided a systematic survey and categorization of tasks, including co-registration, super-resolution, enhancement, classification, segmentation, cross-modality, brain network analysis, and brain decoding. This paper also highlighted the challenges and future directions of the latest work with the expectation that future research can be beneficial.

## 1. Introduction

Brain imaging, providing a way to non-invasively map the structure and function of the brain, has developed significantly in recent years (Gui et al., 2010). For instance, functional brain imaging, such as functional magnetic resonance imaging (fMRI), has the potential to revolutionize researchers' understanding of the physical basis of the brain and offers a powerful tool to understand how the brain adapts to various cognitive activities and tasks (Allen et al., 2014). Additionally, it offers a powerful tool that assists in understanding how the brain adapts to various cognitive activities and tasks. Generative artificial intelligence refers to new technologies that employ existing data including images, text, and audio files to create new content. This new content has a similar underlying pattern of real-world data and has great potential applications in many areas. Synthetic data from generative AI (Wang et al., 2017; Lei et al., 2020b) can train machine learning models (Liu Y. et al., 2021; Lei et al., 2022b) to be less biased and help robots to learn more abstract concepts both in the real and virtual world. The development of neuroimaging as a cross-discipline between imaging and neuroscience has enabled the qualitative and quantitative analysis of images in multiple dimensions. Neuroimaging is a powerful tool for studying brain science, revealing the anatomical structure and working mechanisms of the brain, as well as diagnosing and treating brain diseases. The synergistic developments between emerging analytic technologies and data-sharing initiatives have the potential to transform the role of neuroimaging in clinical applications. While basic neuroscience focuses on understanding how brain activity produces behavior, clinical applications aim to develop tools that are useful for clinical decision-making and treatment development.

**Figure 1:**
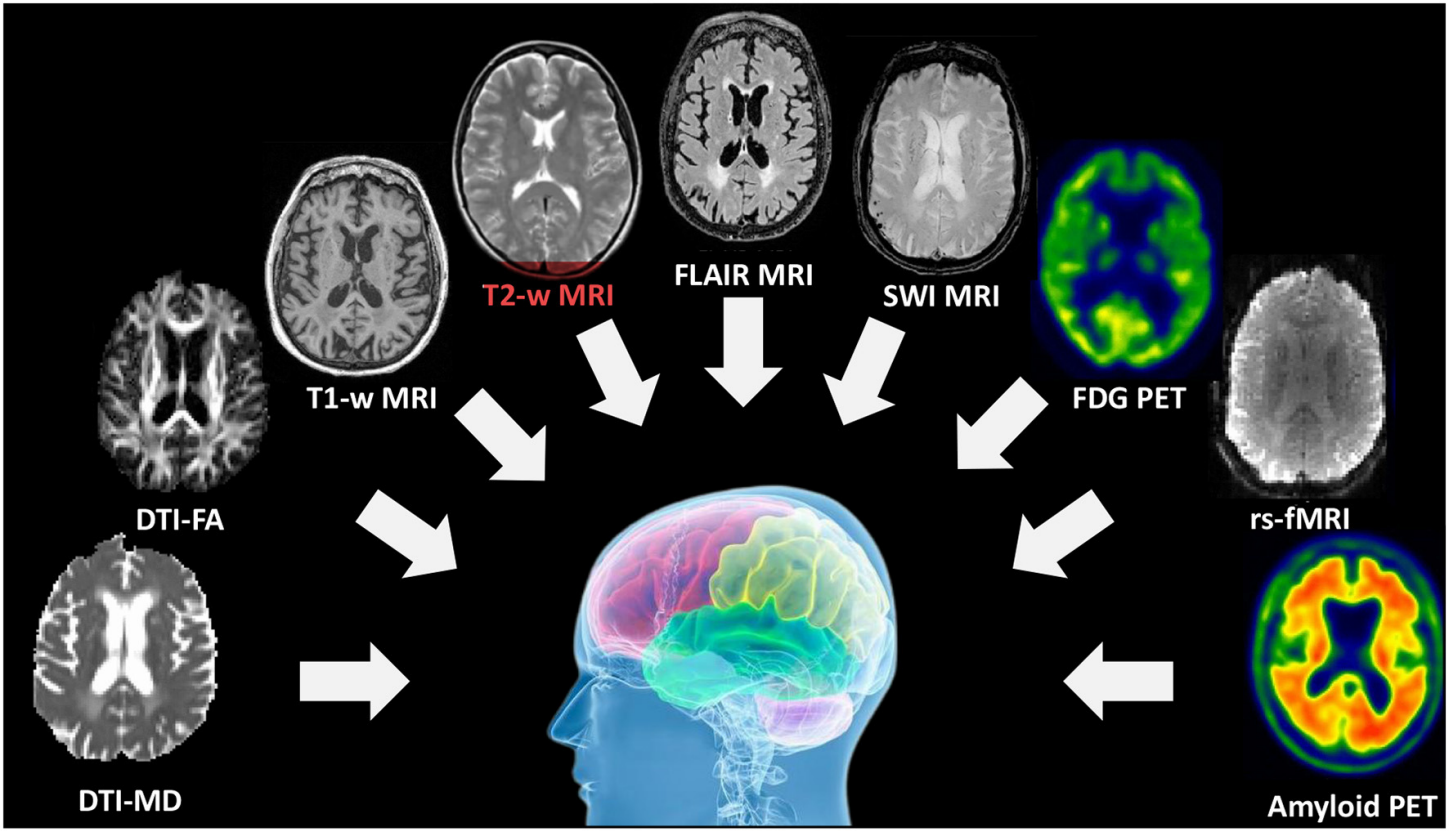
Multi-modality brain imaging includes DTI, sMRI, fMRI, PET, and other imaging types.

Brain images reveal multiple modalities due to different imaging principles and techniques. As shown in Figure 1, multi-modality brain imaging contains many different types, such as diffusion tensor imaging (DTI), fluid-attenuated inversion recovery (FLAIR) MRI, Susceptibility weighted imaging (SWI) MRI, resting state functional MRI (rs-fMRI) and fluorodeoxyglucose positron emission tomography (FDG-PET), etc. Brain imaging can be divided into two broad categories such as functional and structural imaging. Functional neuroimaging, which has generated great optimism about its potential to both revolutionize researchers' understanding of the physical basis of the brain and to provide clinically useful tools (Yu et al., 2021b), has made significant progress in achieving the former goal. However, functional neuroimaging results and models have yet to be incorporated into clinical practice. For decades, numerous translational neuroimaging and radiological studies have identified the characteristics that predict health-related outcomes (Wang S.-Q. et al., 2015; Wang et al., 2020a), including current diagnostic categories and measures of symptoms (Lei et al., 2022a), cognitive and affective computing processes, and cognitive performance. Redefining diagnostic categories, identifying neuropathological features, and assessing healthy brain function outside of current clinical diagnostic categories are potential outcomes of such studies.

Further analysis of brain images can provide morphological information about brain regions, such as their volume, thickness, and surface area. Automated computer analysis has replaced expert anatomists' manual labeling of brain images. In voxel-based morphometry, voxels are segmented into one of three tissue categories (cerebrospinal fluid, white matter, or gray matter) based on their image intensity. After recording all scans in the study into a common anatomical space, the gray matter density of each voxel can be compared between the whole brain and the subject, using the average brain as a template. This process extracts graphical data information about brain-related patterns from brain imaging voxels with high-resolution structures. Lundervold and Lundervold (2019) demonstrated that the introduction of automated computer analysis of magnetic resonance imaging has facilitated the *in vivo* study of whole-brain coordinated patterns in thousands of individuals (Hu et al., 2020b).

Moreover, in the field of network neuroscience (Bassett and Sporns, 2017), the theory and applications of generative AI offer a powerful tool for brain imaging and brain network computing including but not limited to extraction of brain spatiotemporal features and the reconstruction of the topological connectivity of brain networks (Calhoun et al., 2014; Gong et al., 2022). Brain networks, which represent the global connectivity of the brain's structure and function, are crucial in understanding the neural basis of cognitive processes, neuroanatomy, functional brain imaging, and neurodevelopment. Brain network computing involves the construction, reconstruction, analysis, and optimization of brain networks. While brain imaging allows for the qualitative and quantitative analysis of the brain's anatomical and functional structure in two or three dimensions, brain network computing enables the study of brain topological features and covariant features (Isallari and Rekik, 2021). Various tools, such as PANDA (Cui et al., 2013) and GRETNA (Wang J. et al., 2015), can be used for constructing brain networks. However, the brain networks produced by these tools are subjective, time-consuming, and depend on the operator's experience. This review also surveys the development of AI-based algorithms that can automatically construct brain networks.

This paper provides a brief overview of research related to generative learning models in brain imaging from three different perspectives: AI-based generative models, tasks for brain imaging, and the prospects of generative AI for brain imaging. The paper reviews recent developments and advancements made in each of these areas.

## 2. Generative learning model

**Figure 2:**
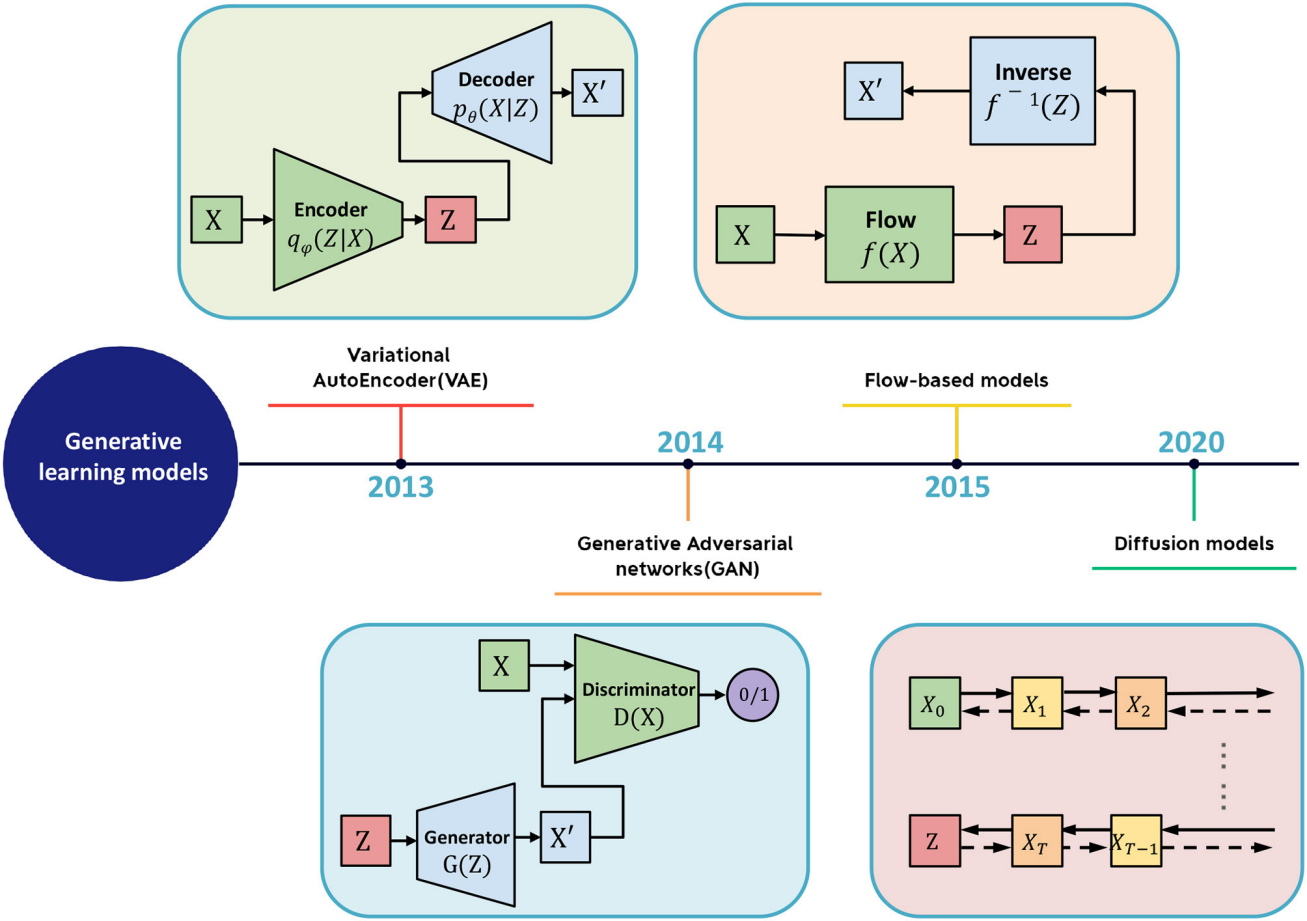
The schematic diagram of generative learning model includes VAE, GAN, flow-based model, and diffusion model.

The generative learning model refers to a class of machine learning (ML) models that can generate new data similar to the training data on which they were trained. Large-scale generative models are trained on massive datasets and require specialized hardware, such as GPUs, for efficient training. As shown in Figure 2, several types of generative models exist, including Generative Adversarial Networks (GANs), Variational Autoencoders (VAEs), Flow Models, and Denoising Diffusion Probabilistic Models (DDPMs). Introduced by Goodfellow et al. (2014), GANs are a type of neural network comprising two parts: a generator network that creates new data and a discriminator network that distinguishes between real and fake data. VAEs, proposed by Kingma and Welling (2013), generate new data by learning a compressed representation of the input data. Flow Models, proposed by Rezende and Mohamed (2015), model the probability distribution of the input data and invert it. DDPMs, a new type of generative model introduced by Ho et al. (2020), have gained popularity in recent years. They draw inspiration from the physical process of gas molecule diffusion, in which molecules diffuse from high-density to low-density areas. DDPMs learn to model the data distribution from input data incrementally.

### 2.1. Variational autoencoder

Autoencoders are a type of neural network that encode the input *X* into a low-dimensional vector *z*, also known as the latent space, and then reconstruct the input *X* based on *z*. By minimizing the error between *X* and the generated output $\hat{X}$, autoencoders are trained to gradually reduce the reconstruction error, thereby achieving the goal of reconstruction. However, autoencoders suffer from the limitation of not being able to generate new content as they cannot produce latent vectors arbitrarily. This is because the latent vectors *z* are all encoded from the original images by the encoder.

In Equation (1), where a set of raw data samples *X*_1_, ..., *X*_*n*_ is available to describe the population, direct sampling from the probability distribution *p*(*X*) would be feasible if *p*(*X*) were known. However, in practice, the distribution of the raw data *p*(*X*) is typically unknown.


(1) $$\begin{array}{l} \;p\left(X\right)=\underset{Z}{\sum }p\left(X|Z\right)p\left(Z\right) \end{array}$$

(2) $$\begin{array}{l} p(Z)=\sum_{X}p(Z|X)p(X)\\ \;=N(0,I)\sum_{X}p(X)\\ \;=N(0,I) \end{array}$$

To address this issue, researchers have added constraints to the latent space *Z* (the space corresponding to the latent vectors) to impose a prior distribution on the latent vectors. This led to the development of the variational autoencoder (VAE) model, which adds a constraint to the encoder to force it to produce latent variables that follow a normal distribution in Equation (2). It is this constraint that distinguishes VAE from traditional autoencoders.

A key aspect of variational autoencoders (VAEs) is the addition of a constraint that enforces a normal distribution on the latent space *Z*. Determining this normal distribution is the primary objective of VAEs. To specify a normal distribution, two parameters must be determined: the mean μ and the standard deviation σ. To accomplish this, the encoder encodes input samples *X* into two latent dimension vectors, μ and σ, which represent the mean and variance of the latent space assumed to follow a normal distribution (Mo and Wang, 2009). To sample *Z* from this latent space, VAE assumes that the latent normal distribution can generate the input images. VAE first samples a random vector ϵ from the standard normal distribution *N*(0, *I*), and then computes:


(3) $$\begin{array}{l} Z=\mu +\sigma ⊙\epsilon \end{array}$$

Here ⊙ denotes element-wise multiplication. *Z* is a vector sampled from the latent space, and *Z* is used as input to the decoder to generate a reconstructed sample $\hat{X}$. The above steps constitute the forward propagation process of the entire network. To perform backpropagation, the loss function is evaluated from two aspects: the similarity between the generated output $\hat{X}$ and the original input *X* and the similarity between the distribution of the latent space and the normal distribution. The similarity between *X* and $\hat{X}$ is generally measured using reconstructions loss, while the similarity between two distributions is generally measured using the Kullback-Leibler divergence (Joyce, 2011).


(4) $$KL(p(x)∥q(x))=\int^{\text{​}}p(x)ln\frac{p(x)}{q(x)}dx$$

(5) $$\begin{array}{l} Loss(X,\hat{X})=Loss_{reconstruction}\\ \;+\beta Loss_{\text{KL}}(z,N(0,I_{d})) \end{array}$$

In summary, VAEs have found useful applications in brain imaging. VAEs can effectively cluster similar patterns in brain activity data and detect subtle changes that may not be easily perceptible to the human eye (Tezcan et al., 2017; Cheng et al., 2021). Furthermore, the learned lower-dimensional representation by VAEs can also serve as a data compression method to minimize computational resources when analyzing complex brain network data (Qiao et al., 2021). The ability to model complex data and generate new data points that resemble the original ones makes VAEs a powerful tool in gaining insights into the underlying mechanisms of neurological disorders and diseases (Zhao et al., 2019).

### 2.2. Generative adversarial network

A Generative Adversarial Network (GAN) is a type of machine learning framework designed by Goodfellow et al. (2014). GANs are composed of two neural networks that compete against each other in a zero-sum game, where one agent's gain is another agent's loss. The framework learns to generate new data with the same statistics as the training set, enabling the GAN to generate new data that resembles the original data.


(6) $$\begin{array}{l} \underset{G}{\min}\;\underset{D}{\max}V\left(D,G\right)w=E_{x\sim p_{\text{data}}\left(x\right)}\left[\log D\left(x\right)\right]\\ \;+E_{z\sim p_{z}\left(z\right)}\left[\log\left(1-D\left(G\left(z\right)\right)\right)\right] \end{array}$$

GANs are based on game theory and can be viewed as a two-player minimax game, where the generator aims to minimize the difference between the distribution of the generated samples and the distribution of the real data, while the discriminator aims to maximize the difference between the two distributions. During training, the generator tries to produce samples that can mislead the discriminator into thinking they are real, while the discriminator tries to correctly distinguish between real and synthetic samples.

The generator usually consists of a series of deconvolutional layers that gradually upsample the random input vector into a sample that is intended to resemble the training data. The discriminator usually consists of a series of convolutional layers that downsample the input image or sample into a lower-dimensional feature representation, followed by a few fully connected layers that compute the final prediction. The loss function used in GANs is typically the binary cross-entropy loss, which measures the difference between the predicted probabilities of the discriminator and the true labels (0 for synthetic samples and 1 for real samples). Other loss functions such as Wasserstein distance or hinge loss have been proposed to address some of the limitations of the binary cross-entropy loss. However, the original GAN suffers from the issue of gradient vanishing, which can lead to unstable training and poor sample quality. One of the main challenges of training GANs is the mode collapse problem, where the generator produces a limited variety of samples that are similar to each other, rather than generating diverse samples that cover the entire range of the training data. Several techniques have been proposed to overcome this problem, such as adding noise to the input of the discriminator, using different types of regularization, or using multi-scale or multi-modal architectures. To overcome this limitation, methods such as WGAN were introduced, which use the Wasserstein distance to measure the distance between the real and generated distributions.


(7) $$W_{c}[\tilde{p}(x),q(x)]=\underset{\gamma \in \Pi (\tilde{p}(x),q(x))}{\inf}E_{(x,y)\sim \gamma }[c(x,y)]$$

The Wasserstein GAN (WGAN), proposed by Arjovsky et al. (2017), aims to overcome the limitations of the original GAN model by using the Wasserstein distance to measure the distance between the real and generated distributions. The objective of WGAN is to minimize the optimal transport cost function, which represents the minimum cost of transforming the generated distribution *q*(*x*) into the real distribution $\tilde{p}\left(x\right)$ through a series of small steps. The cost of each step is measured by the cost function *c*(*x, y*), which represents the distance between the samples *x* and *y*. By using the Wasserstein distance instead of the Jensen-Shannon divergence used in the original GAN, WGAN is able to provide more stable training and generate higher quality samples.


(8) $$\underset{G}{\arg\;\min}\underset{T,\|T{\|}_{L}\leq 1}{\arg\;\max}E_{x\sim \tilde{p}(x)}[T(x)]-E_{x\sim q(z)}[T(G(z))]$$

WGAN, which uses the Wasserstein distance instead of the Jensen-Shannon divergence used in the original GAN, provides more stable training and generates higher quality samples. WGAN also has other advantages, such as improved convergence properties and the ability to measure the distance between distributions more accurately. Overall, WGAN represents a significant advancement in the field of generative modeling and has been successfully applied in various applications, such as image generation, data augmentation, and domain adaptation. Its success has led to the development of several variants, such as Wasserstein GAN with Gradient Penalty (WGAN-GP; Gulrajani et al., 2017), which further improves the stability and efficiency of training.

Another widely used GAN variant in the medical field is CycleGAN, a type of unsupervised learning technique proposed by Zhu et al. (2017), which can learn the mapping between two different domains without any paired data. CycleGAN has several advantages, such as its ability to learn the mapping between two domains without the need for paired data and its ability to handle multimodal and many-to-many mappings. It has been successfully applied in various applications, including medical image analysis, such as image-to-image translation, segmentation, and registration. CycleGAN has also inspired the development of several variants, such as DualGAN (Yi et al., 2017), DiscoGAN (Kim et al., 2017), and UNIT (Liu et al., 2017), which further improve the performance and versatility of the original CycleGAN. The main idea behind CycleGAN is to use two generators and two discriminators to learn the mapping between the domains. The formula for CycleGAN is as follows:


(9) $$\begin{array}{l} G^{*},F^{*}=\arg\;\underset{G,F}{\min}\;\underset{D_{x},D_{Y}}{\max}ℒ(G,F,D_{X},D_{Y}) \end{array}$$

The two generators in CycleGAN are used to generate images from one domain and then transform them into images from the other domain. The two discriminators are used to distinguish between the generated images and the real images from the other domain. The CycleGAN objective function includes two GAN losses, which encourage the generators to generate realistic images, and a cycle-consistency loss, which encourages the generators to learn a mapping between the two domains.


(10) $$\begin{array}{l} ℒ(G,F,D_{X},D_{Y})={ℒ}_{\text{GAN}}(G,D_{Y},X,Y)\\ \;+{ℒ}_{\text{GAN}}(F,D_{X},Y,X)\\ \;+\lambda {ℒ}_{\text{cyc}}(G,F) \end{array}$$

(11) $$\begin{array}{l} {ℒ}_{\text{GAN}}(G,D_{Y},X,Y)=E_{y\sim_{P\text{tan}}(y)}[\log D_{Y}(y)]\\ \;+E_{x\sim_{P\text{tan}}(x)}[\log(1-D_{Y}(G(x))] \end{array}$$

(12) $$\begin{array}{l} {ℒ}_{\text{cyc}}(G,F)=E_{x\sim p\text{tan}(x)}[\|F(G(x))-x{\|}_{1}]\\ \;+E_{y\sim p\text{tan}(y)}[\|G(F(y))-y{\|}_{1}] \end{array}$$

Overall, GANs have found useful applications in brain imaging and network analysis. They can generate synthetic data samples that resemble real data (Dar et al., 2019), enabling researchers to explore brain activity patterns and identify underlying structures. GANs can also augment data by generating synthetic samples to balance imbalanced classes in the dataset, improving deep learning performance in tasks such as image segmentation and classification (Gao et al., 2021). Also, GANs (Lei et al., 2020a) can generate new brain activity patterns (Zuo et al., 2021) in brain network analysis, which can be used to simulate brain activity under various conditions and understand how the network responds to different stimuli. GANs (Wang et al., 2020b) can also help model the relationships between different brain regions and predict the functional connectivity patterns of the brain.

### 2.3. Flow-based generative model

Flow-based generative models are a type of deep generative model that can learn to generate new samples similar to a given dataset. Flow-based models are based on the concept of normalizing flows, which are transformations that can map a simple distribution (e.g., Gaussian) to a more complex distribution (e.g., the distribution of the training data). Flow-based models have been applied to a wide range of applications, such as image generation, video generation, text generation, and even molecular design. Several variations of flow-based models have been proposed, such as conditional flow-based models, which can generate samples conditioned on a given input, and autoregressive flow-based models, which can generate samples by sequentially generating each dimension of the sample.

Flow-based models consist of a series of invertible transformations that map a simple distribution to the distribution of the training data. The inverse of each transformation is also computable, which allows for efficient computation of the likelihood of the data and generation of new samples. The transformations can be learned using maximum likelihood estimation or other methods.


(13) $$\begin{array}{l} G^{*}=argmax_{G}\sum_{i=1}^{m}logP_{G}(x^{i})\\ \;\approx argmin_{G}KL(P_{data}∥P_{G}) \end{array}$$

(14) $$\log q(x)=-\frac{D}{2}\log(2\pi )-\frac{1}{2}{\left\|f(x)\right\|}^{2}+\log\left|\det\left[\frac{\partial f}{\partial x}\right]\right|$$

During training, the flow-based model learns to maximize the likelihood of the training data, which is typically computed using the change of variables formula and the likelihood of the simple distribution. The model can be trained using stochastic gradient descent or other optimization methods. It has several advantages over other types of generative models, such as explicit likelihood computation, efficient sampling, and the ability to perform exact inference. However, they also have some limitations, such as the requirement of invertible transformations, which can restrict the expressiveness of the model.

In summary, Flow-Based Generative Models offer a promising approach for modeling complex data distributions and have potential applications in brain imaging and brain network research. These models can accurately cluster brain activity patterns, identify the structure of the data, and generate synthetic data that resemble the real samples (Dong et al., 2022). Additionally, Flow-Based Models can be used to learn a lower-dimensional representation of the functional connectivity patterns in the brain, enabling researchers to identify relevant features for predicting network changes. The direct modeling of likelihood and the ability to generate novel samples make these models a powerful tool in understanding the underlying mechanisms of complex systems.

### 2.4. Diffusion model

Diffusion models belong to the category of latent variable models in machine learning that utilize Markov chains and variational inference to discern the underlying structure of a dataset. They offer a promising avenue for deep generative modeling owing to their straightforward training process, robust expressive capacity, and ability to generate data via ancestral sampling without the prerequisite of a posterior distribution.


(15) $$\begin{array}{l} \log p(x)\geq E_{q\left(x_{1:T}|x_{0}\right)}\left[\log\frac{p\left(x_{0:T}\right)}{q\left(x_{1:T}|x_{0}\right)}\right]\\ \;=\underset{\text{reconstruction term }}{\underset{︸}{E_{q\left(x_{1}|x_{0}\right)}\left[\log p_{\theta }\left(x_{0}|x_{1}\right)\right]}}\\ \;-\underset{\text{prior matching term }}{\underset{︸}{E_{q\left(x_{T}-1|x_{0}\right)}\left[D_{\text{KL}}\left(q\left(x_{T}|x_{T-1}\right)\|p\left(x_{T}\right)\right)\right]}}\\ \;-\sum_{t=2}^{T}\underset{\text{denoising matching term }}{\underset{︸}{E_{q\left(x_{t}|x_{0}\right)}\left[q\left(x_{t-1}|x_{t},x_{0}\right)p_{\theta }\left(x_{t-1}|x_{t}\right)\right]}} \end{array}$$

The optimization of the diffusion model culminates in training a neural network to predict the original image from any time step of the noise image as input, with the optimization objective being to minimize the prediction error. Moreover, the optimization of the noise-matching term in equation 15 can be approximated by minimizing the expected prediction error at each time step using random sampling.


(16) $$\begin{array}{l} L_{0}=-\log p_{\theta }\left(x_{0}|x_{1}\right) \end{array}$$

$$\begin{array}{l} L_{t-1}=D_{KL}\left(q\left(x_{t-1}|x_{t},x_{0}\right)|p_{\theta }\left(x_{t-1}|x_{t}\right)\right) \end{array}$$

$$\begin{array}{l} L_{T}=D_{KL}\left(q\left(x_{T}|x_{0}\right)|p\left(x_{T}\right)\right) \end{array}$$

(17) $$\underset{\theta }{\text{arg min}}E_{t\sim U\{2,T\}}\left[E_{q(x_{t}|x_{0})}\left[D_{\text{KL}}(q(x_{t-1}|x_{t},x_{0})\right]\right]$$

In contrast to other deep generative models such as VAE, GAN, and normalizing flow, diffusion models offer unique advantages while overcoming several limitations and challenges. The training of VAEs can be challenging due to the difficulty in selecting the variational posterior, while GANs require an additional discriminator network, and normalizing flow models have limited expressive power. In contrast, diffusion models utilize the diffusion process of data points through the latent space to derive a solution that involves training only a generator with a simple objective function, without the need for training other networks.

In computer vision, diffusion models train neural networks to denoise images blurred with Gaussian noise by learning to reverse the diffusion process. Three examples of generic diffusion modeling frameworks used in computer vision include denoising diffusion probabilistic models, noise-conditioned score networks, and stochastic differential equations. In brain imaging and brain network analysis, diffusion models serve as a valuable tool for estimating the underlying structure of brain function and structure (Chung and Ye, 2022), which is essential for understanding the mechanisms of neurological disorders and diseases (Myronenko, 2018). By modeling the diffusion of data points through the latent space, diffusion models are capable of effectively capturing changes in brain connectivity over time and identifying regions critical to brain structure (Wolleb et al., 2022). Additionally, diffusion models can simulate brain activity under different conditions and predict how the brain network will respond to various stimuli.

Overall, diffusion models provide a valuable approach to modeling latent spaces in various fields, including computer vision, brain imaging, and brain network analysis. By offering an efficient and effective means of estimating the underlying structure of datasets, diffusion models can be a powerful tool for gaining insights into the spatiotemporal dynamics of large and complex systems.

## 3. Tasks for brain imaging and brain network construction

**Figure 3:**
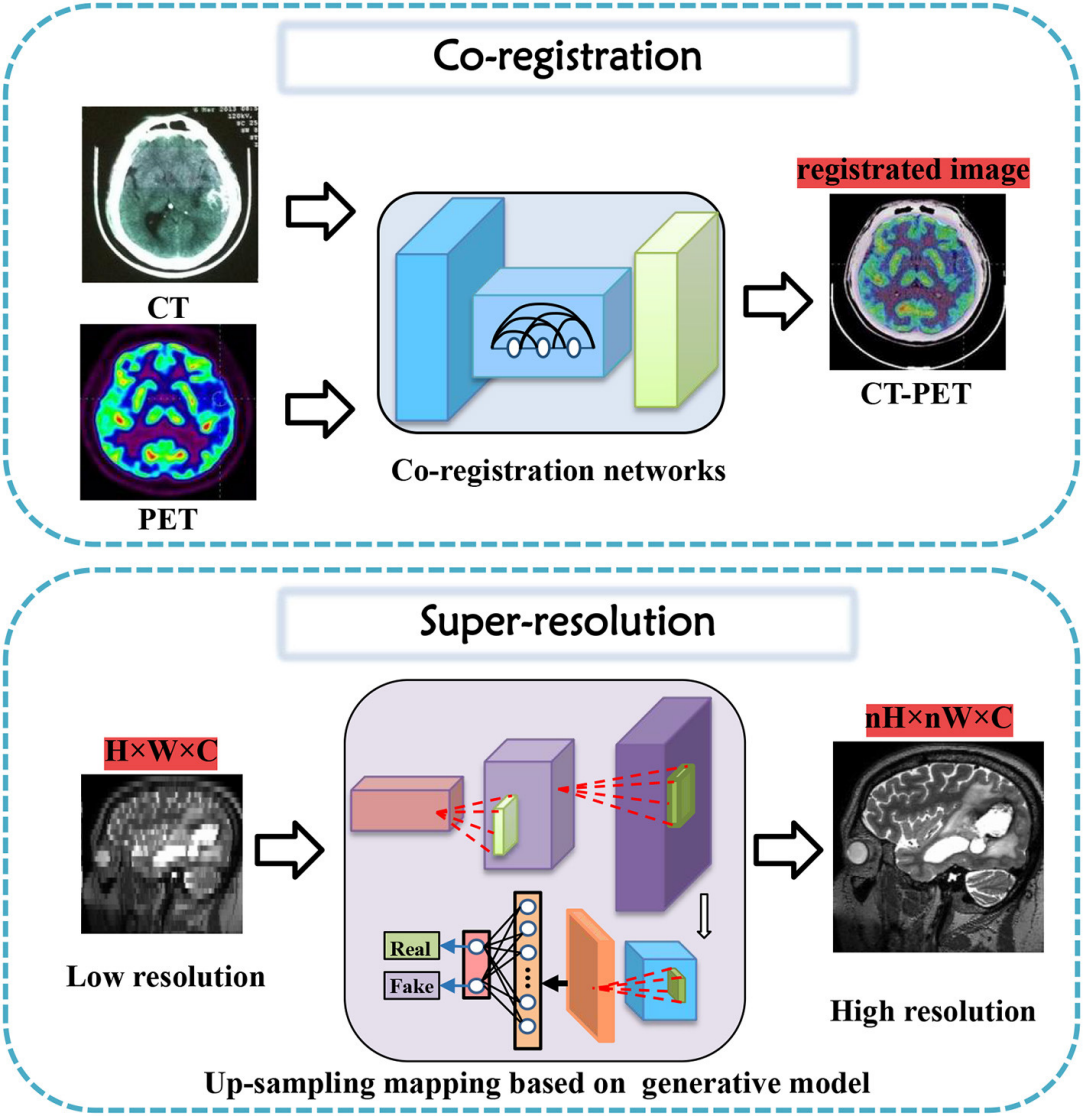
Co-registration and super-resolution task diagrams applying generative AI for brain imaging.

**Figure 4:**
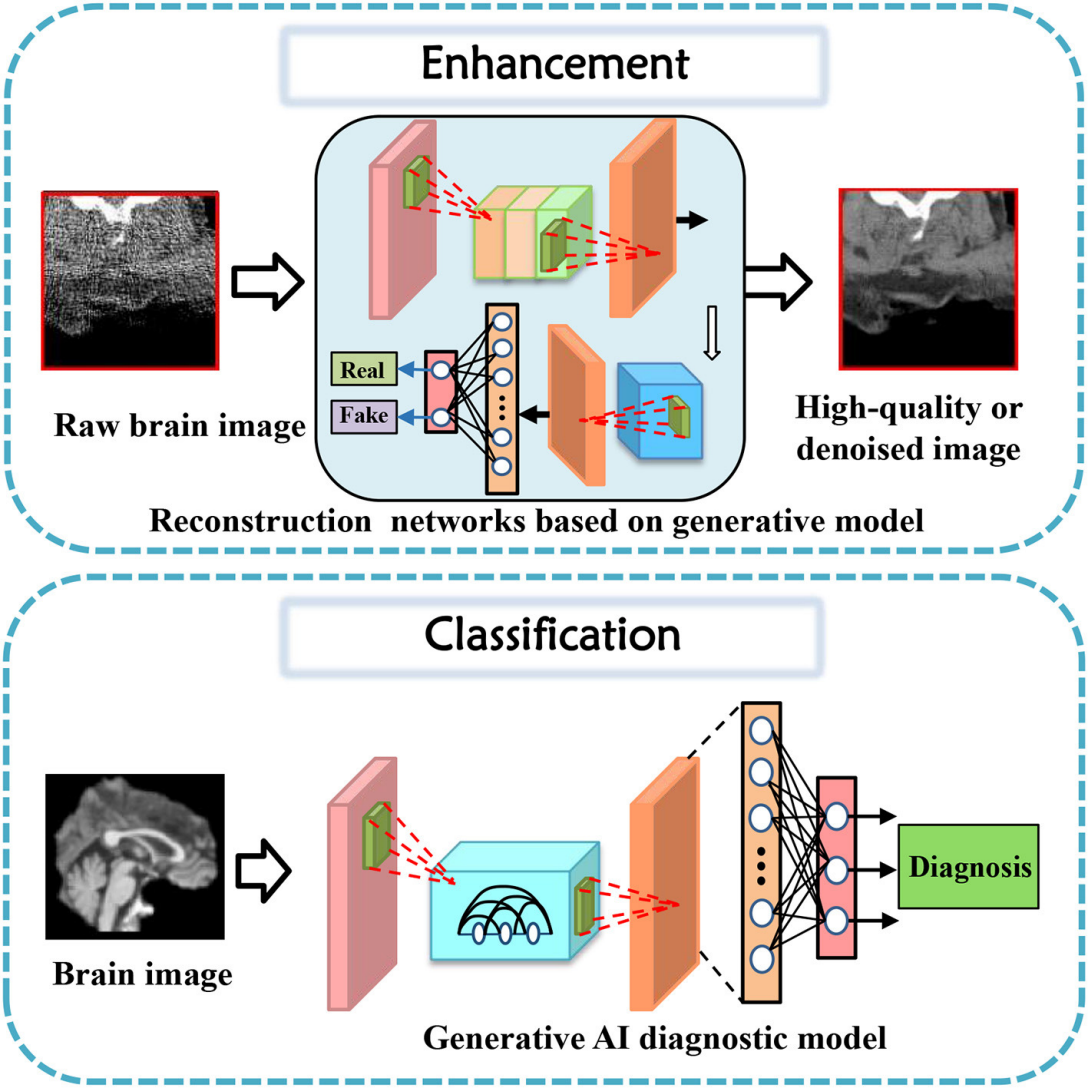
Enhancement and classification task diagrams applying generative AI for brain imaging.

**Figure 5:**
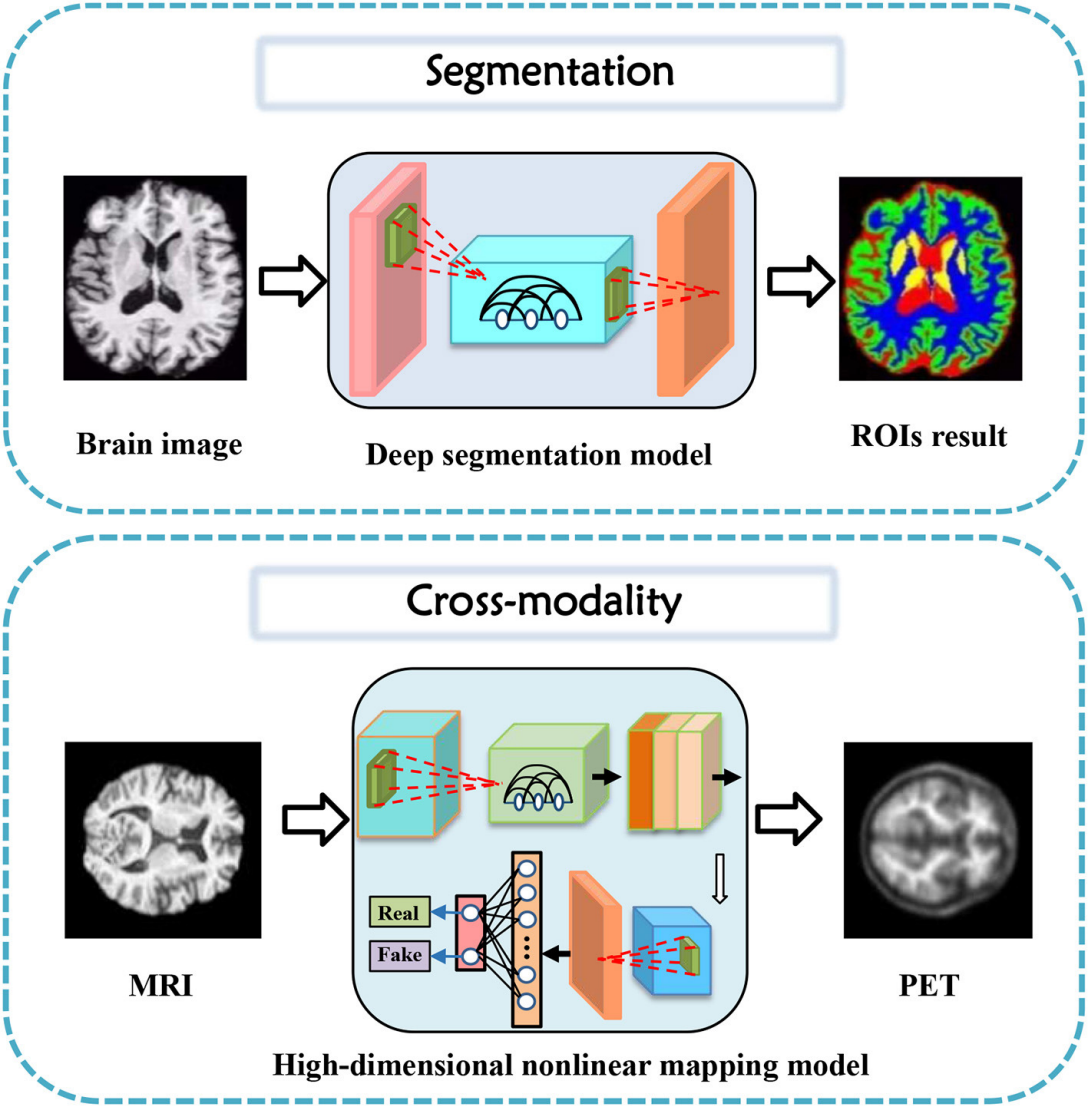
Segmentation and cross-modality task diagrams applying generative AI for brain imaging.

**Figure 6:**
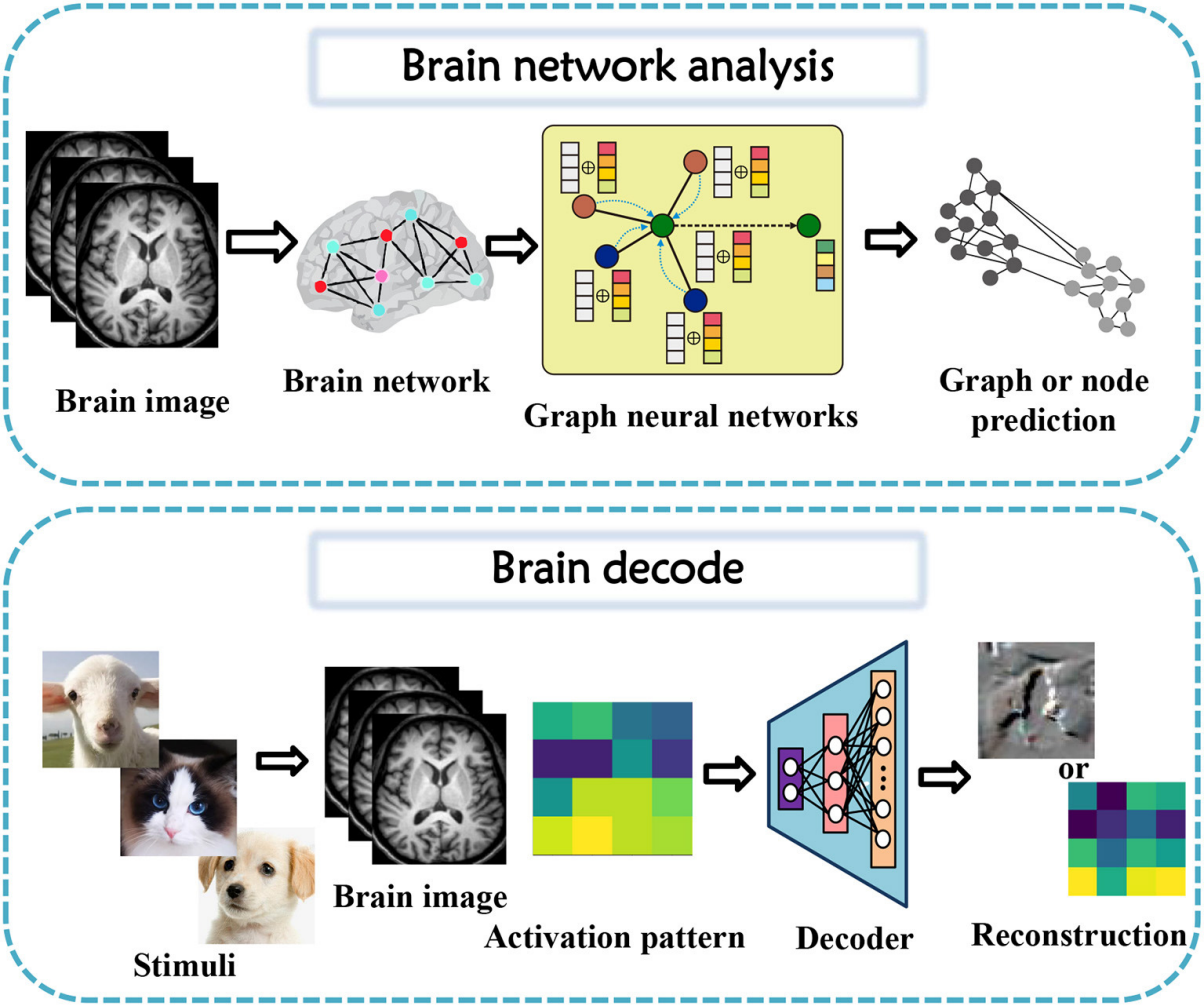
Brain network analysis and brain decode task diagrams applying generative AI for brain imaging.

In this section, tasks in brain imaging and brain network construction are specifically categorized and investigated in eight categories, including co-registration and super-resolution (shown in Figure 3), enhancement and classification (shown in Figure 4), segmentation and cross-modality (shown in Figure 5), and brain network analysis and brain decode (shown in Figure 6).

### 3.1. Co-registration

Co-registration is a crucial step in medical image analysis to align images from different modalities or time points. However, it is a challenging task due to various factors, such as noise, artifacts, motion, and anatomical differences. Many innovative methods have been proposed to tackle these challenges and improve co-registration performance.

For instance, Sundar et al. (2021) proposed conditional GANs to address intra-frame motion problems in dynamic PET studies of the brain. Yang et al. (2020) proposed indirect multimodal image registration and completion by using synthetic CT images obtained from multi-contrast MRI. Kong et al. (2021) introduced RegGAN for image-to-image translation and registration which includes noise reduction. Furthermore, Wang B. et al. (2022) proposed invertible AC-flow for direct generation of attenuation-corrected PET images without CT or MR images. Apart from these deep learning-based methods, a diffusion-based image registration method called DiffuseMorph was introduced by Kim et al. (2022). This method overcomes the limitations of traditional and deep learning-based methods due to computational complexity and topological folding.

These proposed methods have shown promising results in improving co-registration performance in medical imaging. Future research can explore further advances to overcome the remaining challenges, such as reducing the time taken for co-registration while maintaining high accuracy and improving the robustness and generalization of existing solutions.

### 3.2. Super-resolution

Research into high-resolution brain imaging has yielded promising results, with generative models proving to be a popular and effective approach (Sun et al., 2022). One such approach, as described in Song et al. (2020), involves using a GAN architecture with anatomical and spatial inputs for creating super-resolved brain PET images. According to the authors, the proposed GAN outperforms other deep learning models and penalized deconvolution techniques. Similarly, You et al. (2022) suggests using fine perceptive generative adversarial networks (FP-GANs) for high-resolution magnetic resonance imaging. This technique applies a sub-band generative adversarial network and sub-band attention for super-resolution in individual sub-bands.

These studies contribute to the growing body of literature on super-resolution tasks for brain imaging, with generative models emerging as promising solutions. The success of these models suggests that they could be applied to other high-resolution imaging tasks requiring greater detail and precision (Wicaksono et al., 2022). However, further research is needed to fully evaluate the performance and potential limitations of generative models for these tasks.

### 3.3. Enhancement

Data enhancement is a widely adopted approach in improving the performance of deep learning models for various medical image analysis tasks. Some studies cater to the task of data enhancement in medical tasks using generative AI. The first CycleGAN-based method for MR-to-CT synthesis proposed by Wolterink et al. (2017), shows that this technique can generate high-quality synthetic CT scans that are similar in appearance to real ones. Both the designed GAN model and novel loss function take enhancement tasks a step further in their performance (Dar et al., 2019).

Similarly, Yurt et al. (2021) proposed a multi-stream approach that integrates multiple source images to synthesize missing multi-contrast MRI images, outperforming other state-of-the-art methods. Zhan et al. (2021) proposed a Multi-scale Gate Mergence based GAN model that accurately diagnoses patients with corrupted image sequences by weighing different modalities across locations. Luo et al. (2021) proposed an edge-preserving MRI image synthesis GAN model, infusing an auxiliary edge image generation task to help preserve edge information and improve latent representation features, and an iterative multi-scale fusion module to further improve the quality of the synthesized target modality. Recently, Upadhyay et al. (2021) proposed a robust GAN-based framework that models an adaptive loss function to improve robustness to out-of-distribution (OOD)-noisy data and estimates per-voxel uncertainty in predictions for image-to-image translation across two real-world datasets in medical imaging applications. Luo et al. (2022) proposed an adaptive rectification-based GAN model with spectral constraint to synthesize high-quality standard-dose PET images from low-dose PET images, reducing radiation exposure while maintaining accurate diagnoses.

These studies demonstrate the promising potential of deep learning-based approaches and data enhancement in enhancing the quality and performance of medical image generation tasks.

### 3.4. Classification

Classification of brain diseases is a crucial task for early diagnosis and effective treatment. The advancements in deep learning techniques have led to the development of various generative models for the automatic classification of neuroimages. Recently, serval generative models were proposed to focus on brain disease classification tasks.

In 2019, Pan et al. (2019) propose a unified deep learning framework to jointly perform image synthesis and disease diagnosis using incomplete multi-modal neuroimaging data. The proposed method includes two networks: a Disease-Image Specific Neural Network (DSNN) to capture the spatial information of MRI/PET scans and a Feature-consistent Generative Adversarial Network (FGAN) to synthesize missing images by encouraging DSNN feature maps of synthetic images and their respective real images to be consistent. The method achieves state-of-the-art performance for Alzheimer's disease identification and mild cognitive impairment conversion prediction tasks. Besides, pattern expression offered complementary performance to biomarkers in predicting clinical progression, making these deep-learning-derived biomarkers promising tools for precision diagnostics and targeted clinical trial recruitment. Yang Z. et al. (2021) applied deep learning framework to longitudinal data and revealed two distinct progression pathways that were predictive of future neurodegeneration rates.

Indeed, there are several generative models that have been designed with a deeper consideration of prior settings for tasks such as biomarkers and clinical reports. Wang et al. (2020a) propose an ensemble of 3D convolutional neural networks (CNNs) with dense connections for automatic diagnosis of Alzheimer's disease (AD) and mild cognitive impairment (MCI). The proposed model was evaluated on the ADNI dataset using a probability-based fusion method that combines multiple architectures. Shin et al. (2020) propose a GAN-based approach for the diagnosis of Alzheimer's Disease (AD) using T1-weighted MRIs as input data. The authors incorporate AD diagnosis into the training objective to achieve better classification performance. This architecture shows state-of-the-art results for three- or four-class classification tasks involving MCI, normal cognition, or Alzheimer's disease. Kim et al. (2020) propose a GAN-based model for classifying Alzheimer's disease (AD) and normal cognitive condition (NC). The authors use slice-selective learning to reduce computational costs and extract unbiased features. The researchers trained the model using an 18F-fluorodeoxyglucose ([18F] FDG) PET/CT dataset obtained from the Alzheimer's Disease Neuroimaging Initiative database. The approach seems feasible when there are insufficient datasets available for training individual deep neural networks (Wang et al., 2018; Yu et al., 2020) with single-source training datasets.

Furthermore, like Baur et al. (2020) propose a practical method for unsupervised brain MRI anomaly detection in clinical scenarios using a CycleGAN-based style-transfer framework. The proposed approach involves mapping real healthy data to a distribution with lower entropy and suppressing anomalies by filtering high-frequency components during training. The experiments demonstrate that the proposed method outperforms existing methods on various metrics, such as F1 score, PRC AUC, and ROC AUC, thus demonstrating its potential for practical applications in clinical settings.

Therefore, generative models have demonstrated their potential in various classification tasks for brain diseases. These models can extract features that are not directly visible, thereby aiding in the early diagnosis and accurate classification of diseases.

### 3.5. Segmentation

Generative models have gained significant attention in the field of medical image segmentation for their capability of reducing the dependence on manually labeled data. This paper reviewed the recent advances in generative models for segmentation tasks, focusing on brain tumor segmentation (Myronenko, 2018). In 2019, Yuan et al. (2020) presented a 3D unified generative adversarial network, achieving any-to-any modality translation and multimodal segmentation through a single network based on the anatomical structure. Ding et al. (2021) proposed ToStaGAN, a two-stage generative adversarial neural network, for brain tumor segmentation, which incorporates coarse prediction maps with fine-grained extraction modules and dense skip connections. In 2022, Wang S. et al. (2022) introduced Consistent Perception Generative Adversarial Network (CPGAN), an alternative to deep learning algorithms with expensive labeled masks, demonstrating superior segmentation performance over other methods with less labeled data on Anatomical Tracings of Lesions After Stroke. Wu et al. (2021) presented an unsupervised brain tumor segmentation method called Symmetric-Driven Generative Adversarial Network (SD-GAN) in 2021, which utilizes inherent anatomical variations by learning a non-linear mapping between left and right brain images. SD-GAN outperforms state-of-the-art unsupervised methods, providing a promising solution to unsupervised segmentation tasks.

These studies demonstrate that generative models have become increasingly important for medical image segmentation owing to their ability to learn from unannotated data and promising performance in comparison to traditional supervised methods.

### 3.6. Cross-modality

Cross-modality image synthesis has become an active research area in medical imaging, where the goal is to generate images in a target modality from the input in another modality. Several generative models have been proposed for this task, including variations of generative adversarial networks (GANs) and encoder-decoder models. In recent years, significant progress has been made in using generative models for cross-modality image synthesis in the brain.

One of the early works in this area introduced gEa-GAN and dEa-GAN by Yu et al. (2019), which integrated edge information to bridge the gap between different imaging modalities. The resulting synthesized images showed superior quality compared to several state-of-the-art methods, as demonstrated on various datasets. Another study (Hu et al., 2021) introduced Bidirectional GAN, which used a bidirectional mapping mechanism to embed diverse brain structural features into the high-dimensional latent space. The method achieved better quality in generating PET images than other models trained on the same dataset, while preserving diverse details of brain structures across different subjects. Other studies explored more challenging scenarios, such as Jiao et al. (2020) generating magnetic resonance (MR)-like images directly from clinical ultrasound (US) images of fetal brains and Sharma and Hamarneh (2019) synthesizing multiple modalities of neuroimaging data. The former study proposed an end-to-end trainable model that utilized shared latent features between US and MR data to generate realistic MR-like images, while the latter study introduced a multi-input, multi-output variant of GAN to synthesize sequences missing in brain MRI scans. The proposed models achieved promising results and demonstrate the feasibility of using generative models in clinical practice. Moreover, a bidirectional mapping mechanism is designed to embed the semantic information of PET images into the high-dimensional latent potential space for improving the visual quality of the cross-modal synthesized images (Hu et al., 2019, 2020a). The most attractive part is that the method can synthesize perceptually realistic PET images while preserving the different brain structures of different subjects.

Several other studies have also proposed novel generative models, including MouseGAN (Yu et al., 2021c) for segmenting mouse brain structures in MRI images and SC-GAN (Lan et al., 2021) for synthesizing multimodal 3D neuroimaging data. The former study achieved improved segmentation using modality-invariant information, while the latter used spectral normalization, feature matching, and self-attention modules to stabilize the training process and ensure optimization convergence. These studies have shown that generative models have the potential to improve existing neuroimaging analysis tasks and provide new tools for diagnosis and follow-up.

Finally, some studies have attempted to integrate generative models with disease diagnoses (Yang H. et al., 2021). Moreover, in neuroimaging data, One study (Pan et al., 2022) proposed a disease-image-specific deep learning framework that utilizes image-disease specificity to highlight different disease-relevant regions in the brain, with promising results on Alzheimer's Disease and mild cognitive impairment conversion prediction tasks. These studies highlight the potential of generative models to not only generate images of other modalities but also aid in downstream analysis and diagnoses using inter-modality information.

### 3.7. Brain network analysis

Brain network modeling is a critical research field in neuroscience that aims to understand the complex relationships among structural and functional connectivity patterns in the human brain. In recent years, deep learning has been increasingly used in brain network analysis as it shows promising results in predicting brain graphs, inferring effective connectivity, and diagnosing Alzheimer's disease using multimodal neuroimaging data. To this end, several deep learning frameworks have been proposed to generate reliable individual structural connectivity from functional connectivity, MultiGraphGAN, and MGCN-GAN proposed by Bessadok et al. (2020) and Zhang et al. (2020). These frameworks combine adversarial learning and topology preservation to generate high-quality brain graphs from limited data effectively. Some studies focused on inferring effective connectivity from functional MRI data, including EC-GAN and MGCN-GAN proposed by Liu et al. (2020), incorporate adversarial learning and graph convolutional networks to effectively infer brain structure-function relationships. Additionally, some studies aimed at diagnosing Alzheimer's disease using multimodal neuroimaging data, such as MRL-AHF and HGGAN proposed by Zuo et al. (2021) and Pan et al. (2021), which leverage adversarial learning and hypergraph representation to effectively integrate and represent multimodal data. Overall, deep learning frameworks have been shown to hold great potential in brain network modeling, rapidly advancing our understanding of brain structure-function relationships and improving the prediction accuracy of brain disorders, such as brain drug addiction (Gong et al., 2023). Future research in this area will likely continue to explore and develop new deep learning-based approaches to further enhance modeling accuracy and generalization performance.

### 3.8. Brain decoding

Generative models have become a popular research focus in the field of brain decoding tasks, especially in the reconstruction of perceived images from fMRI signals. Baek et al. (2021) proposed a hierarchical deep neural network model of the ventral visual stream to explain the innate emergence of face-selectivity. VanRullen and Reddy (2019) applied a deep learning system to reconstruct face images from fMRI data, achieving accurate gender classification and decoding of visually similar inputs. Ren et al. (2021) proposed the Dual-Variational Autoencoder/Generative Adversarial Network framework, which outperforms state-of-the-art methods in terms of visual reconstruction accuracy. Chen et al. (2022) introduced the MinD-Vis framework, which uses a self-supervised representation of fMRI data and a latent diffusion model to reconstruct high-quality images with semantic details, outperforming state-of-the-art methods in semantic mapping and generation quality. Dado et al. (2022) presented a novel experimental paradigm, HYPER, for neural decoding of faces from brain recordings using generative adversarial networks, achieving the most accurate reconstructions of perception to date. These studies demonstrate the potential of generative models in brain decoding tasks, which can help advance our understanding of brain function and perception.

**Figure 7:**
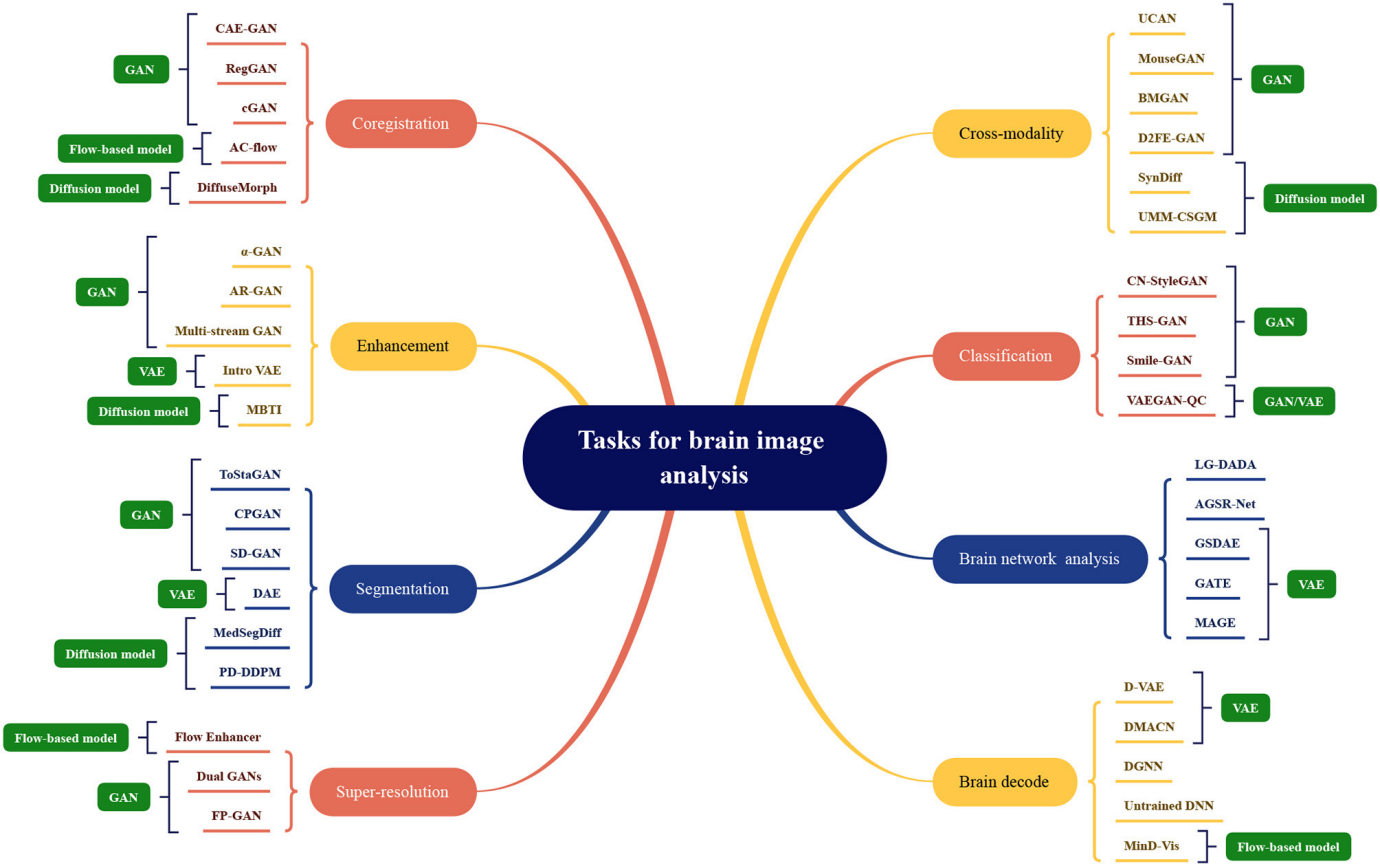
Model categorization map under different brain image analysis tasks. Coregistration: CAE-GAN (Yang et al., 2020), RegGAN (Kong et al., 2021), cGAN (Sundar et al., 2021), AC-flow (Wang B. et al., 2022), DiffuseMorph (Kim et al., 2022); Enhancement: α-GAN (Kwon et al., 2019), AR-GAN (Luo et al., 2022), Multi-stream GAN (Yurt et al., 2021), Intro VAE (Hirte et al., 2021), MBTI (Rouzrokh et al., 2022); Segmentation: ToStaGAN (Ding et al., 2021), CPGAN (Wang S. et al., 2022), SD-GAN (Wu et al., 2021), DAE (Bangalore Yogananda et al., 2022), MedSegDiff (Wu et al., 2022), PD-DDPM (Guo et al., 2022); Super-resolution: Flow Enhancer (Dong et al., 2022), Dual GANs (Song et al., 2020), FP-GAN (You et al., 2022); Cross-modality: UCAN (Zhou et al., 2021), MouseGAN (Yu et al., 2021c), BMGAN (Hu et al., 2021), D2FE-GAN (Zhan et al., 2022), SynDiff (Özbey et al., 2022), UMM-CSGM (Meng et al., 2022); Classification: CN-StyleGAN (Lee et al., 2022), THS-GAN (Yu et al., 2021a), Smile-GAN (Yang Z. et al., 2021), VAEGAN-QC (Mostapha et al., 2019); Brain network analysis: LG-DADA (Bessadok et al., 2021), AGSR-Net (Isallari and Rekik, 2021), GSDAE (Qiao et al., 2021), GATE (Liu M. et al., 2021), MAGE (Pervaiz et al., 2021); Brain decode: D-VAE (Ren et al., 2021), DMACN (Lu et al., 2021), DGNN (VanRullen and Reddy, 2019), Untrained DNN (Baek et al., 2021), MinD-Vis (Chen et al., 2022).

The application division of generative artificial intelligence methods in the field of brain image analysis is shown in Figure 7. The existing models mentioned above are divided according to tasks, and the names of the corresponding models are marked under the relevant tasks, which can be mainly divided into eight categories as shown in the figure. The most representative methods shown in the figure, the more research is optimized under this mainstream model. The four mainstream methods have different adaptation conditions for different tasks. In terms of coregistration, cross-modality, segmentation, classification, clustering, and super-scoring tasks, the optimization of the GAN model is significantly better than the other three mainstream models. The reason is that the GAN-based method can be well applied to brain image generation tasks. In brain network analysis and brain decoding tasks, the encoding and decoding structure of the VAE-based method will have more advantages. Flow-based models have relatively few applications and have a certain degree of application in super-resolution, brain decoding, and co-registration tasks. Diffusion models have high-quality generation effects and have been gradually used in various tasks of brain image analysis, and have achieved certain achievements.

## 4. Discussion

### 4.1. Challenge

The domain of brain image analysis and brain network computing confronted many obstacles that have impeded the development of the field. In order to progress future research more effectively, this review delves into some significant challenges and expounds on them in the following:

#### 4.1.1. Small sample problem

Medical image datasets are generally much smaller than datasets in other fields, due to the challenging task of acquiring and annotating medical images. For example, for lung nodule detection tasks, due to the small number of lung nodules, the number of positive samples in the dataset is very small, and the size, shape, and position of lung nodules also vary greatly, making it difficult for algorithms to accurately detect lung nodules. Therefore, the small sample problem has become one of the major obstacles for machine learning algorithms in the field of medical imaging. In addition to methods such as meta-learning that can effectively solve the small sample problem, improvement can be done by using prompts to modify pre-trained models, utilizing prior knowledge, and model ensembles. For example, in lung nodule detection tasks, pre-trained models can be used to extract features and prompts can be used to guide the model on how to detect lung nodules. In addition, prior knowledge can be used to constrain the output of the model, such as constraining the size and shape of the output lung nodules. Finally, model ensembles can be used to improve algorithm robustness and generalization capabilities.

#### 4.1.2. High dimensional data problem

Medical scans or images are typically high-dimensional data types that contain large amounts of information. For example, in brain medical imaging, 3D MRI or fMRI is typically used to obtain brain structure and functional information. These data often contain millions of pixels or thousands of time points, so extracting meaningful, non-redundant, and non-overfitting features from such high-dimensional data is a new challenge for machine learning algorithms in the field of medical imaging. For the task of feature extraction from high-dimensional brain data, due to the complexity of brain structure and function, single modality feature extraction methods often have difficulty in extracting meaningful features. For example, in brain MRI images, tissue such as gray matter, white matter, and cerebrospinal fluid have different shapes and positions, so multi-modality feature analysis methods are required to extract meaningful features. In addition, as there are complex relationships between brain structure and function, multi-modality feature analysis methods are necessary to extract the relevant features between structure and function.

#### 4.1.3. Realism of different modalities

Generative models have been widely applied in the field of medical data generation. However, the generated modality data may suffer from the problems of unreliability or modality inconsistency. MRI and fMRI are common modalities in medical imaging. For MRI image generation, there may be problems such as insufficient reconstructed image resolution, lacking local details, and artifacts. In fMRI data generation, there may be signal suppression in local regions, interference from noise, and motion artifacts. In recent years, the generation of genetic data has also had a significant impact on the medical field. One major problem in generating genetic data is that the generated data may differ from real genetic data because real genetic data are produced by many genetic factors working together. Thus, when generating genetic data, multiple factors must be considered to improve the realism and consistency of generated data. While problems that could occur in extensively experimented image modalities may be easily identified and optimized, this realism problem can be hard to discern in complex or abstract modalities. Ultimately, such erroneously generated data may lead to incorrect diagnoses or treatments. To address this problem, researchers have proposed many methods, such as introducing special loss functions, such as cycle consistency in the model process in the image domain, and introducing strategies such as multi-modality joint and multi-task learning to improve the generative quality and realism of modal data.

#### 4.1.4. Standard validation issues

The lack of universally accepted validation standards for evaluating the performance of machine learning models in the field of medical imaging makes it difficult to compare results from different studies or ensure the best performance of the models. As the structure of the human brain is complex and diverse, specific to the generation task of brain diseases, more requirements are raised, and large differences exist between different regions, thus how to evaluate the performance of generative models to produce meaningful generative models. For example, for the generation task of MRI images, evaluation metrics can use traditional image quality evaluation indicators such as PSNR, SSIM, FID, etc., but these indicators cannot fully reflect the performance of the model in medical applications, such as whether the model generates anatomy structure consistent with real data, and whether it can better display lesion areas. Therefore, researchers have proposed some specific evaluation indicators, such as similarity of structure with real images, neuron activation, diagnostic accuracy, etc., to more accurately evaluate the performance of generative models. However, the universality and comparability of these indicators still need more experimental verification and exploration in order to be better applied to different tasks and datasets.

#### 4.1.5. Model interpretability

The issue of model interpretability is another challenge that must be addressed in medical image generation models. The model needs to have a certain level of interpretability so that physicians and researchers can understand the model's predictions and generate results. In the task of generating brain disease, model interpretability is particularly critical. Doctors need to be able to understand the relationship between the abnormal structures in the generated images and the underlying diseases, in order to make accurate diagnoses and treatment decisions. During the generation of images, the model may introduce factors such as image noise and artifacts, which can seriously affect medical diagnosis. To address these issues, interpretability techniques can provide valuable assistance. For example, visualization techniques can help doctors and researchers better understand the generated results and identify abnormal factors by generating comparative images and visualizing the internal feature maps of the model. In addition, model interpretability can also be achieved by adding interpretation layers or using interpretable models. However, there are some limitations to the use of data in the scenario of generating brain diseases. For example, due to privacy issues involving patients, medical imaging data is often highly sensitive and therefore difficult to obtain large-scale datasets directly. In addition, the different brain structures of different patients pose a challenge to the generated results of the model. Therefore, when generating brain diseases, it is necessary to consider the balance between data usage scenarios and model interpretability in order to obtain more accurate and interpretable results.

#### 4.1.6. Limitation

There are still inescapable limitations to brain imaging computing using generative AI. Firstly, before the application of the synthetic brain image data set for training, if the differences between the synthetic data set and the real data set are not fully studied, the generated results will be biased. Secondly, most current generative brain image analysis methods may generate illogical “unnatural data” due to the lack of labels and causal features in the generating process. The robustness and reliability of the algorithm may be impacted. Thirdly, in the process of model training, it is possible to remember the distribution of original training samples. If the original training sample can be reversely inferred from the synthesized data, there will be “implicit privacy” leakage problem, and how to protect privacy more closely is still a question to be explored.

### 4.2. Future direction

New generative methods in brain network research are likely to find applications in both basic and clinical research. In the coming years, generative learning and signal processing techniques will remain essential tools for furthering our understanding of the brain. This paper presents three perspectives on future approaches to brain network research:

#### 4.2.1. Brain circuit identification

The application of generative models in brain circuit research is gaining more attention. Through the utilization of generative models, researchers are able to extract significant information about neural circuits from brain data. In the field of neuroscience, neural circuits are key elements for understanding brain function and are crucial for regulating various cognitive and affective behaviors. Deep learning can assist in revealing the intricate structure, profound functionality, and impact of the brain. Generative models can support neural circuit research by learning data features in brain circuits. In particular, generative models can be employed to generate, transform, and improve neuroimaging data, consequently creating novel and high-quality data to facilitate a more profound comprehension of neural circuits. Furthermore, generative models can serve as a data augmentation technique to diversify the limited neural circuit data samples, which can boost both training and disease diagnosis efficiency. Moreover, this technique can simulate and generate experiments related to the connection and variation of neural circuits among patients, ultimately resulting in more experimental evidence and predictive capacity for neural circuit research, therefore yielding more information for disease diagnosis and treatment.

#### 4.2.2. Precise localization of brain regions

In the field of brain disorders, exploration of potential treatment options is a common practice. Generative models are capable of assisting in the comprehension of neural regulation and localization by producing intricate images that reflect the interconnectivity between different regions of the brain. Neural regulation denotes the procedure through which the brain controls behavior and emotions by moderating the excitatory and inhibitory activities of neurons. In neuroscience, generative models can simulate and forecast complex neural regulation processes. These models are instrumental in comprehending the mechanisms of neural regulation during brain development, deducing the reciprocal interaction between neurons and synapses, and predicting the patterns of connectivity between distinct neurons. Furthermore, synthetic neural imaging data is useful in providing researchers with a better appreciation of macroscopic neural regulation patterns. To pinpoint the location and function of particular brain areas, researchers can input significant image data into a generative model to learn the features of brain structures and more precisely localize brain regions. In functional connectivity analysis, generative models can generate hypothetical functional connectivity data and compare it with actual data to identify the links between various brain regions. The identification of possible targets for the treatment of brain-related disorders involves investigating the associations between the functions of neurons, synapses, and specific diseases. Generative models, as tools for data analysis and prediction, can effectively learn features and patterns automatically from vast amounts of neural data, and aid in target identification for the moderation or treatment of such diseases. Thus, generative models can enable the comprehension of neural regulation and brain localization, facilitate the search for targets and solutions to treat brain disorders, and ultimately improve patients' quality of life.

#### 4.2.3. Brain diseases and mechanisms

Neurodegenerative diseases, particularly Alzheimer's disease, are caused by damage to neurons and synapses in the brain, resulting in declines in cognitive and memory function and eventually leading to dementia. Studies have shown that generative models can forecast the speed of a patient's cognitive function decline, aiding physicians in early disease diagnosis. These models can also assimilate prior knowledge on diverse physiological, neural, cognitive, and behavioral processes, such as visual information processing, perception, language, and memory cognition. Generative models have helped in analyzing the onset mechanism of the disease and developing individualized treatment plans. Consequently, they are expected to become pivotal tools for exploring and comprehending brain mechanisms, with the potential to boost the precision of neurodegenerative disease diagnosis and treatment (Jing et al., 2022).

#### 4.2.4. Multi-scale brain atlas

Population-based multi-scale brain research is a prominent focus in neuroscience (Betzel and Bassett, 2017). Its goal is to integrate information from multiple levels to understand the structure and function of the brain, establish connections between them, and gain insights into the working principles of the brain. Macro-level research investigates the overall structure and function of the brain, while micro-level research focuses on the cellular-level structure and function of neurons and synapses. Meso-scale research investigates small structures, such as cortical columns, connections, and neuronal clusters. Genomics studies the influence of genes on the brain's structure and function. Generative models integrate multiple data sources to reveal the complex mechanisms of the brain and explore the interactions between neurons and brain regions, providing a comprehensive view of the brain network. They can also predict gene expression data, diagnose and treat individual differences in diseases. Although the use of generative models in the study of the brain is still in its early stages, the accumulation of data and technological advances are expected to expand their usage.

These approaches will help build a comprehensive and accurate representation of the human brain and enable the discovery of new insights across neurological and psychiatric disorders.

## 5. Conclusion

This article provides a review of generative artificial intelligence for brain image computing and brain network computing. Generative AI can be divided into four main methods: variational autoencoder (VAE), generative adversarial network (GAN), flow-based model, and diffusion model. These models offer a promising solution for analyzing and interpreting large-scale brain imaging data. Generative AI has enabled researchers to gain a better understanding of the brain's physical basis and how it adapts to various cognitive activities in the field of brain imaging. In the context of brain network computing, generative AI can be used to reconstruct the topological connectivity of brain networks. However, there are limitations associated with using generative AI for analyzing brain imaging data. For instance, medical imaging data is often highly sensitive due to privacy issues involving patients, making it difficult to obtain large-scale datasets directly. Additionally, different brain structures of different patients pose a challenge to the generated results of the model. Therefore, when using generative AI techniques to generate brain diseases or analyze large-scale medical imaging datasets, it is necessary to balance data usage scenarios and model interpretability in order to obtain more accurate and interpretable results. In conclusion, generative AI has broad application prospects in brain imaging and brain network, which can help to better understand the internal function and structure of the brain, promote the diagnosis and treatment of brain diseases, and provide new opportunities and methods for neuroscience research.

## Author contributions

SW and YH proposed the idea and co-managed the project. YH, SW, and CG co-designed the framework. CG, CJ, XC, CP, GH, SW, and YH co-wrote the manuscript. MN and AS supported the project and offered resources to accomplish this research. All authors read, contributed to revision, and approved the manuscript.
